# Editorial: Metal organic frameworks for antimicrobial prevention and treatments

**DOI:** 10.3389/fchem.2025.1636763

**Published:** 2025-06-16

**Authors:** Maria Cristina Cassani, Raymond J. Turner, Francesca Bonvicini, Silvia Panzavolta

**Affiliations:** ^1^ Department of Industrial Chemistry “Toso Montanari”, University of Bologna, Bologna, Italy; ^2^ Department of Biological Sciences, University of Calgary, Calgary, AB, Canada; ^3^ Department of Pharmacy and Biotechnology, University of Bologna, Bologna, Italy; ^4^ Department of Chemistry “G. Ciamician”, University of Bologna, Bologna, Italy

**Keywords:** MOFs, antimicrobial activity, antibacterial photodynamic activity, zinc imidazolate framework, zinc(II) complexes, curcumin, aminocinnamic acid, titanium scaffolds

We have entered what is now referred to as the antimicrobial resistance (AMR) era, where resistance to existing antibiotics and antiseptics by bacteria has become increasing the norm. Unfortunately, the development of new antibiotics has declined as the rates of resistance increased. The WHO and the UN has defined this as the hidden pandemic and the greatest threat to human health and food security. To address this problem alternative approaches to traditional antibiotics are being explored that include but not limited to, antimicrobial peptides, bacteriophage, resistance inhibitors, vaccines, probiotics, natural plant compounds, CRISPR vectors etc. One approach has been to revisit metal elements, which were used as antimicrobials since antiquity and forgotten about with the discovery of antibiotics. Metal elements provide multifactorial killing on microbes (Lemire et al., 2013). The mode of delivery of metallo-antimicrobials may be as the metal salt, an alloy, organometallics, complexes, nanomaterial and of increasing interest, metal-organic frameworks (MOFs) (Turner, 2023).

MOFs have been explored for drug delivery, bioimaging and sensing (Nguyen et al., 2024; Linnane et al., 2022), but they are also being explored as antimicrobials (Shen et al., 2020; Li et al., 2021). Some properties of MOFs that are appealing as antimicrobials include the ability to control or stimulate decomposition, they can interact well and specifically with bacterial membranes, they can be designed towards photo/chemical catalysis generating reactive oxygen species, or as in the drug delivery designed for high loading and defined release capacities for wound treatments. Another exciting potential of MOFs is to explore synergistic possibilities through combining an organic antibiotic as the ligand and an antimicrobial element as the metal.

In this Research Topic Zinc and Titanium were the main characters of the collection. A popular Zn-based MOF such as zeolitic imidazolate framework 8 (ZIF-8) has been the subject of two contributions either as coating or as a carrier for drug delivery.

In the first case described by Di Matteo et al., ZIF-8 was used for coating porous Ti6Al4V scaffolds, either bare or previously modified using hydroxyapatite (HA) or HA and gelatin (HAgel), via a growing single-step method in aqueous media using two contact times at 6 h and 24 h. In clinical orthopedics, the bone implant-related infections caused by bacterial adhesion on the surface of the implants are considered a serious issue, and the systemic administration of antibiotics may not guarantee the effective removal of bacteria from the site of the infection. The materials described in this work proved to be suitable for bone regeneration, mimicking a physiological environment for osteoblast proliferation and cytocompatible, according to proliferation analyses. Moreover, the presence of ZIF-8 on the surface of the differently coated scaffolds, produced following a deposition time of 24 h, allowed for an excellent antibacterial function of the biomaterials. In this context, ZIF-8-modified Ti scaffolds perfectly meet this requirement as bacterial adhesion to the biomaterials was completely inhibited within the 4-h time frame of the anti-adhesion assay, and bacterial proliferation was completely arrested up to 8 h of incubation as results of the highest release of Zn^2+^ ions in the surrounding environment. This is of clinical relevance because the orthopedic implants are most susceptible to bacterial colonization in this post-surgery time window.

The natural photosensitizing properties of curcumin (Cur) are widely acknowledged however, its limited bioavailability has impeded its practical application. In the second case dealing with ZIF-8, Shang et al. have developed a nanomaterial called Cur@ZIF-8@BA by encapsulating curcumin (Cur) within ZIF-8 and modifying the surface with boric acid (BA). The Cur@ZIF-8@BA exhibits pH-responsive properties and enhances bacterial binding, thereby effectively promoting photodynamic therapy. Furthermore, Cur@ZIF-8@BA exhibits long-term physiological stability and low toxicity. Its antibacterial activity against *Escherichia coli*, *Staphylococcus aureus* and *Acinetobacter baumannii* is significantly increased in the presence of light compared to a dark environment. The mechanism behind this may be that BA increases the affinity of Cur@ZIF-8@ BA towards bacteria and making released Zn^2+^ and BA from the nanomaterial increase bacterial cell membrane permeability. This facilitates efficient delivery of Cur into bacterial cells, resulting in generation of abundant reactive oxygen species (ROS) and subsequent bactericidal activity.

d’Agostino et al., reported the synthesis, through solid-state and solution methods, of a novel series of coordination compounds that involve the combination of the mild antimicrobials 3- and 4-aminocynnamic acids with the zinc(II) metal ion. Antimicrobial activity for all compounds was tested against the reference strains of the pathogenic bacteria *Pseudomonas aeruginosa*, *Staphylococcus aureus*, and *Escherichia coli*. The newly synthesized Zn-compounds demonstrated equal or superior antimicrobial activity compared to the parent compounds and, unexpectedly, they also exhibited anti-biofilm activity. We posit that integrating the antimicrobial attributes of organic molecules with those of metal ions in coordination compounds may signify advancements in this endeavor. Nevertheless, these initial strides underscore the need for thorough contemplation to comprehend the mechanisms through which co-crystals and coordination polymers act against bacteria. Ongoing studies demanding additional specialized and collaborative research are currently underway to delve deeper into these aspects.

Finally in the work of Altharawi et al., a novel titanium/1,3,5-Tris(4-carboxyphenyl)benzene metal-organic frameworks (Ti/BTBMOFs) was synthesized by using titanium nitrate and 1,3,5-Tris(4-carboxyphenyl)benzene (BTB) under microwave radiation. The *in vitro* anticancer properties of Ti/BTB-MOF were evaluated using the MTT method against MG-63/bone cancer cells and A-431/skin cancer cells. In the anticancer activity, IC_50_ (half-maximal inhibitory concentration) values of 152 μg/mL and 201 μg/mL for MG-63/bone cancer cells and A-431/skin cancer cells, respectively, were observed. In the antibacterial activity, minimum inhibitory concentrations (MICs) of 2–64 μg/mL were observed against studied pathogenic strains. The antimicrobial activity of Ti/BTB-MOF was higher than that of penicillin and gentamicin. Therefore, the synthesized Ti/BTB-MOF could be introduced as a suitable bioactive candidate.

A barrier of moving forward with MOFs as antimicrobials is the various drug administrative agencies which do not have defined paths of approval/regulations for such combined compounds. This is in part burdened by not having a clear idea of their biological mechanisms or their pharmokinetics (Alavijeh et al., 2018) as research in this avenue is severely lacking compared to the discovery of new MOFs with antimicrobial activities.
